# Zirconium-Based MOFs and Their Biodegradable Polymer Composites for Controlled and Sustainable Delivery of Herbicides

**DOI:** 10.1021/acsabm.2c00499

**Published:** 2022-07-29

**Authors:** Lila A. M. Mahmoud, Richard Telford, Tayah C. Livesey, Maria Katsikogianni, Adrian L. Kelly, Lui R. Terry, Valeska P. Ting, Sanjit Nayak

**Affiliations:** † School of Chemistry and Biosciences, University of Bradford, Bradford BD7 1DP, United Kingdom; ‡ School of Pharmacy, Al-Zaytoonah University of Jordan, Amman 11733, Jordan; § Polymer IRC, Faculty of Engineering and Informatics, University of Bradford, Bradford BD7 1DP, United Kingdom; ∥ Bristol Composites Institute, Department of Mechanical Engineering, University of Bristol, Bristol BS8 1TR, United Kingdom

**Keywords:** MOF, composite, zirconium, MCPA, agriculture, pesticide

## Abstract

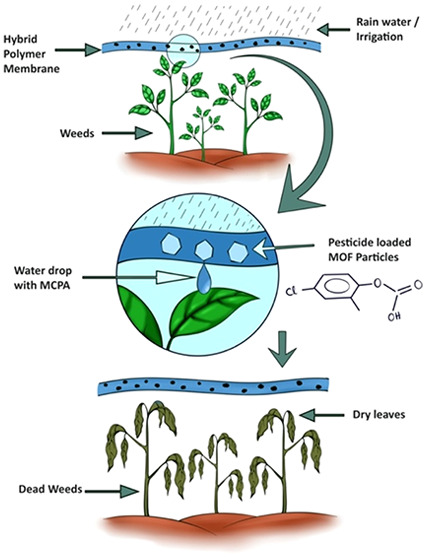

Adsorption and controlled release of agrochemicals has been studied widely using different nanomaterials and a variety of formulations. However, the potential for application of high surface-area metal–organic frameworks (MOFs) for the controlled release of agrochemicals has not been thoroughly explored. Herein, we report controlled and sustainable release of a widely used herbicide (2-methyl-4-chlorophenoxyacetic acid, MCPA) via incorporation in a range of zirconium-based MOFs and their biodegradable polymer composites. Three Zr-based MOFs, viz., UiO-66, UiO-66-NH_2_, and UiO-67 were loaded with MCPA either postsynthetically or in situ during synthesis of the MOFs. The MCPA-loaded MOFs were then incorporated into a biodegradable polycaprolactone (PCL) composite membrane. All three MOFs and their PCL composites were thoroughly characterized using FT-IR, TGA, SEM, PXRD, BET, and mass spectrometry. Release of MCPA from each of these MOFs and their PCL composites was then studied in both distilled water and in ethanol for up to 72 h using HPLC. The best performance for MCPA release was observed for the postsynthetically loaded MOFs, with PS-MCPA@UiO-66-NH_2_ showing the highest MCPA concentrations in ethanol and water of 0.056 and 0.037 mg/mL, respectively. Enhanced release of MCPA was observed in distilled water when the MOFs were incorporated in PCL. The concentrations of herbicides in the release studies provide us with a range of inhibitory concentrations that can be utilized depending on the crop, making this class of composite materials a promising new route for future agricultural applications.

## 1 Introduction

With rising global populations, our ever-increasing demand for food has resulted in the growing increase in the use of pesticides and herbicides, as an essential part of modern agriculture.^1−3^ However, cumulative accumulation of pesticides in the environment, food, and drinking water has been directly linked to health problems and diseases including an increased risk of cancer,^4−7^ with a number of herbicides being banned in recent years for the risks they pose to human health.^8^ In addition, it has been recognized that the toxicity of these agrochemicals is not only confined to human populations, with aquatic and marine ecosystems being severely affected by the increasing accumulation of pesticides in water through agricultural runoff.^9−11^ In order to address this problem, there is an urgent need for the development of cleaner and safer, more controlled technologies for delivering pesticides and other agrochemicals.

A large proportion of the pesticides used in modern farming are applied in such a way that they miss the target vegetation and are thus released into the environment, contributing heavily to the resulting pollution and ecotoxicity.^12^ Therefore, one way to reduce the release of pesticides to the environment is by more controlled and targeted delivery of agrochemicals.^13^ In this study, we are proposing a contact-based approach for delivering herbicides using hybrid composite membranes comprised of metal–organic frameworks (MOFs) in a biodegradable polymer.

**Scheme 1:**
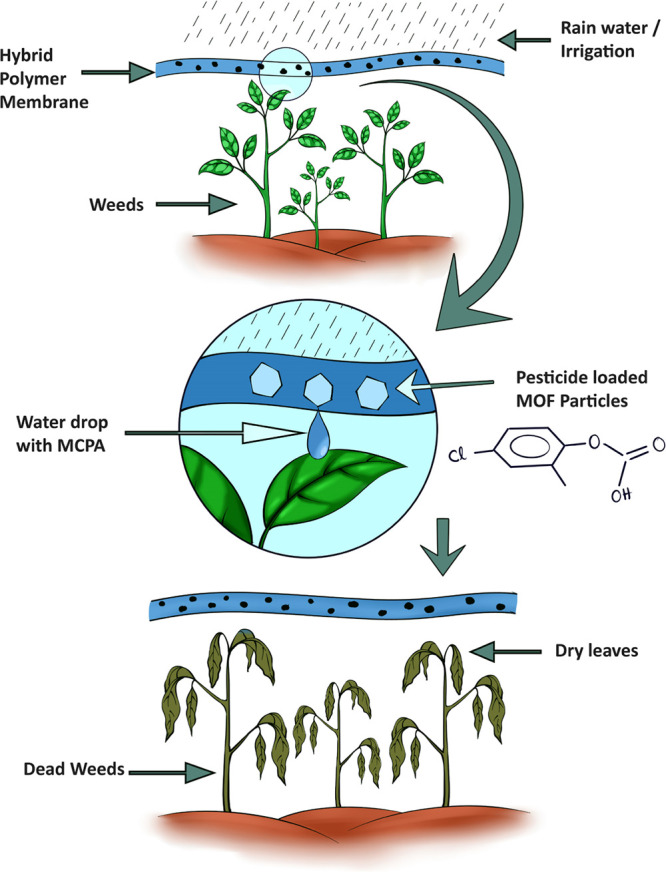
Polymer-MOF Composite Membrane for Delivering Pesticides in Close Contact with Weeds, Facilitated by the Rainwater or Irrigation System, Is Illustrated

MOFs are well-known for their ultrahigh porosity and surface area.^14−16^ Because of their ability to host different kinds of molecules and allow their controlled release, MOFs have been studied extensively for drug delivery applications.^17−20^ Recently, MOFs have been shown to be useful for the separation and sensing of toxic agrochemicals.^21−23^ Similar to their use in controlled drug delivery, MOFs can potentially act as vector for sustainable delivery of pesticides.^24−26^ However, their crystalline powdered or granular form is a barrier to practical applications. Integration of MOFs in polymer composites can potentially solve this problem with the additional benefit of targeted delivery of pesticides when the composite is in direct contact with the plants, with release being assisted by natural rain or water sprinkler/irrigation systems as illustrated in Scheme 1. With a contact-based delivery approach of the pesticides, the hybrid composites will reduce unnecessary runoff of agrochemicals in water and soil, and therefore, can potentially act as an environmentally friendly means of delivering pesticides for the future.

In this study, 4-chloro-2-methylphenoxyacetic acid (MCPA) was used as a model herbicide. MCPA is widely used as a controlling agent for broadleaf weeds, and is a major global contributor to contamination of soil and groundwater, as demonstrated by its ubiquitous presence in drinking water across the world.^27−30^

Herein, we report the loading and release of MCPA using three different Zr-based MOFs:^31,32^ UiO-66, UiO-66-NH_2_, and UiO-67, along with their biodegradable composite polycaprolactone membranes, as a potential future route for delivering pesticides in a more controlled and sustainable way.

## 2 Experimental Section

### 2.1 Synthesis

#### 2.1.1 Materials Used

All reagents were purchased from Sigma-Aldrich, and the solvents were purchased from Fisher Scientific. All chemicals were used without any further purification.

#### 2.1.2 Synthesis of Pristine UiO-66

In a 40 mL Teflon lined glass vial, 0.8985 g (5.408 mmol) of 1,4-benzenedicarboxyllic acid (BDCA) and 0.6300 g (2.703 mmol) of ZrCl_4_ were added followed by addition of 16 mL of dimethylformamide (dmf) and 0.5 mL of conc. HCl. The resulting mixture was sonicated for 20 min at 25 °C and placed in a programmable oven at 120 °C for 24 h with a heating rate of 10 °C per min, followed by cooling to 25 °C at a rate of 2 °C per min. The resulting white solid was filtered using vacuum filtration through a Buchner funnel. The residue was thoroughly washed with fresh dmf and dried under a vacuum to yield a white crystalline solid. Yield: 90%, with respect to Zr. IR (neat, cm^–1^): 1660 (w), 1583 (m), 1507 (w), 1393 (s), 1257 (w), 1158 (w), 1101 (w), 1019 (w), 885 (w), 820 (w), 744 (s), 659 (s). Full IR spectra are shown in Figure S1.

#### 2.1.3 Synthesis of Pristine UiO-66-NH_2_

In a 40 mL Teflon lined glass vial, 0.125 g (0.536 mmol) of ZrCl_4_ was added to 5 mL of dmf and 1 mL of conc. HCl. The solution was then sonicated at room temperature for 20 min. After sonication, 0.134 g (0.740 mmol) of 2-aminoterephthalic acid was added to the vial along with 10 mL of dmf. The mixture was sonicated for another 20 min at room temperature and was then placed in an oven for 24 h at 80 °C at a heating rate of 10 °C per min and a cooling rate of 2 °C per min until it reached room temperature. The resulting pale-yellow precipitate was collected using vacuum filtration through a Buchner funnel and then washed three times with 10 mL of dmf. The dried product, a pale-yellow crystalline solid, was collected. Yield: 89.9% with respect to Zr. IR (neat, cm^–1^): 3464 (b), 3350 (b), 1654 (s), 1569 (s), 1495 (m), 1433 (s), 1385 (s), 1339 (m), 1260 (m), 1158 (w), 1101 (w), 970 (w), 894 (w), 823 (w), 798 (w), 766 (m), 656 (s).

#### 2.1.4 Synthesis of Pristine UiO-67

0.067 g (0.288 mmol) of ZrCl_4_ was added to 0.09 g (0.372 mmol) of 4,4-biphenyldicarbocylic acid (BPDC) in a 40 mL Teflon-lined glass vial followed by addition of 16 mL of dmf and 0.5 mL of conc. HCl. The resulting solution was sonicated at room temperature for 20 min and the vial was then placed in a programmable oven at 80 °C for 24 h, with a heating rate of 10 °C per min and a cooling rate of 2 °C per min until it reached room temperature. The resulting cream-colored precipitate was vacuum filtered through a Buchner funnel and washed three times with dmf and dried under vacuum to yield a white crystalline solid. Yield: 71.3%, with respect to Zr. IR (neat, cm^–1^): 1674 (m), 1606 (m), 1541 (m), 1498 (w), 1407 (s), 1297 (m), 1180 (w), 1101 (w), 1007 (w), 928 (w), 880 (w), 846 (m), 758 (s), 895 (m), and 856 (s). Full IR spectra are shown in Figure S2.

#### 2.1.5 Synthesis of UiO-66 Using MCPA as a Modulator (IS-MCPA@UiO-66)

In a 40 mL Teflon-lined glass vial, 0.898 g (5.405 mmol) of BDCA, 0.630 g (2.703 mmol) of ZrCl_4_, and 2.170 g (10.816 mmol) of MCPA were added followed by addition of 16 mL of dmf. The resulting solution was sonicated for 20 min at 25 °C. The vial was then placed into an oven at 120 °C for 24 h, with a heating rate of 10 °C per minute and a cooling rate of 2 °C per minute until it reached room temperature. The resulting white cloudy suspension was vacuum filtered through a Buchner funnel and washed three times with 10 mL of dmf and dried under vacuum to yield a white crystalline solid. Yield: 32.3%, with respect to Zr. IR (neat, cm^–1^): 1660 (m), 1580 (m), 1504 (w), 1433 (w), 1390 (s), 1254 (w), 1189 (w), 1158 (w), 1095 (w), 1064 (w), 1019 (w), 823 (w), 743 (m), and 655(s).

#### 2.1.6 Synthesis of UiO-66-NH_2_ Using MCPA as a Modulator (IS-UiO-66-NH_2_)

IS-MCPA@UiO-66-NH_2_ was synthesized with the same method as UiO-66-NH_2_, but by using 0.2968 g (1.479 mmol) of MCPA as modulator instead of HCl. The resultant solution was pale yellow and cloudy, the contents of the vial were filtered via vacuum filtration through a Buchner funnel and washed with 10 mL of dmf to yield a crystalline yellow solid. Yield: 12%, with respect to Zr. IR (neat, cm^–1^): 3484 (b), 3376 (b), 1654 (m), 1563 (m), 1493 (w), 1430 (s), 1385 (s), 1339 (m), 1260 (m), 1104 (w), 1061 (w), 968 (w), 763(m), and 661 (s).

#### 2.1.7 Synthesis of UiO-67 Using MCPA as a Modulator (IS-MCPA@UiO-67)

IS-MCPA@UiO-67 was synthesized using the same methods as UiO-67. However, 0.149 g (0.743 mmol) of MCPA was used instead of HCl as a modulator, at a linker to modulator ratio of 1:2. The resultant white precipitate was vacuum filtered through a Buchner funnel and washed three times with dmf to yield a white crystalline solid. Yield: 34.2%, with respect to Zr. IR (neat, cm^–1^): 1666 (w), 1600 (m), 1546 (w), 1495 (w), 1409 (s), 1178 (w), 1098 (w), 1007 (w), 853 (w), 767 (m), 696 (w), 670 (m).

#### 2.18 Activation of MOFs

For the activation of the MOFs, the solids were first washed with dmf, and then dried in an oven at 80 °C for 2 h. The dried MOFs were submerged in methanol for 30 min, filtered, and then dried in an oven before being left in a vacuum oven for 24 h at 115 °C.

#### 2.1.9 Postsynthetic Loading of MCPA (PS-MCPA@UiO-66, PS-MCPA@UiO-66-NH_2_, PS-MCPA@UiO-67)

A stock solution of MCPA was first prepared by dissolving 1.2 g of MCPA in 100 mL of ethanol. In separate glass vials, 0.05 g of each of the activated pristine MOF samples was weighed followed by addition of 10 mL of MCPA solution. The resulting suspensions were stirred for 24 h at 800 rpm. The cloudy suspensions were subsequently centrifuged. The precipitates were washed three times with ethanol (3 × 10 mL) and left in an oven to dry at 80 °C for 4 h.

#### 2.1.10 Preparation of Polycaprolactone-MOF Composites

In a beaker, 0.2 g of polycaprolactone (PCL) pellets were added along with 15 mL of chloroform. The mixture was then left to stir for 30 min until all the PCL pellets were dissolved. Five milligrams of MOF solid was weighed and ground with a mortar and pestle and then 5 mL of the PCL:chloroform mixture was added to the ground MOFs and mixed. The PCL-MOF mixture was then transferred into silicon molds using a pipet and was left to dry overnight. This resulted in thin PCL-MOF composite sheets that could be easily peeled off the molds.

### 2.2 Herbicide Release Studies

Herbicide release studies were performed in ethanol and distilled water by adding 5 mg and 20 mg of the loaded MOFs and polymer-MOF composites, respectively, into 2 mL of each solvent. The MOFs and the polymer-MOF composites were left in the solvents for 72 h, and then the solvent was filtered out and analyzed to quantify MCPA using HPLC (Section 2.3).

### 2.3 Characterization

Fourier-transformed Infrared (FTIR) spectra were recorded over the range of 600–4000 cm^–1^ using a PerkinElmer Spectrum 100 FTIR spectrometer fitted with a PerkinElmer Universal ATR sampling device. Thermogravimetric analyses (TGA) were carried out using a Q5000IR thermogravimetric analyzer (TA Instruments, USA). Samples (ca. 5 mg) were heated in platinum pans from 30 to 600 °C at 5 °C min^–1^ under a nitrogen purge gas flow of 25 mL min^–1^. TA Instruments Universal Analysis 2000 software was employed to analyze the data. SEM images and energy dispersive X-ray (EDX) elemental analysis data were collected using an FEI Quanta 400 E-SEM instrument fitted with an Oxford Xplore30 EDS system. The samples were sputter-coated with gold using an Emitech K550 coating system and the analyses were carried out under a vacuum. Powder X-ray diffraction (PXRD) data were collected at ambient temperature using a Bruker D8 diffractometer with Cu Kα_1,2_-radiation (λ = 0.154018 nm, 1600 W) source. Electrospray ionization mass spectra (ESI-MS) were recorded using a Thermo Orbitrap LTQ (Thermo Fisher Scientific, UK) equipped with an electrospray ionization source operating in negative mode, with a sample dissolved in methanol and injected at 10 μL min^–1^ using the embedded syringe pump. HPLC-UV-MS analysis on the MCPA extracts was performed using a Waters 2690 HPLC equipped with a 996 PDA detector for UV detection in series with a Quattro Ultima Triple Quadrupole mass spectrometer (Waters LLC, USA) for MS detection operating in electrospray positive mode. The chromatography system and mass spectrometer were controlled using *MassLynx* v. 4.1 software. The HPLC method consisted of a Phenomenex 5 μm C_18_ column (2.1 mm × 150 mm held at 40 °C). Samples were injected without further preparation (10 μL). The mobile phase flow rate was 0.2 mL/min and consisted of A, 5 mM aq. ammonium formate, and B, 5 mM ammonium formate in methanol. The HPLC gradient was T = 0.0, A = 90%, B = 10%; T = 1.0, A = 90%, B = 10%; T = 8, A = 10%, B = 90%; and at T = 12, A = 10% and B = 90%. At T = 12.1 min, the system reverted to the starting conditions and was held for 2.9 min to allow the column to re-equilibrate. UV data were recorded between 210 and 300 nm and ESI^–^ MS in SIR mode recording *m*/*z* 199, which corresponds to [M-H]^−^ of MCPA (cone voltage, etc., were optimized prior to analysis by direct infusion of MCPA reference). MCPA levels were determined using an externally standardized approach from calibration series created using MCPA reference solutions prepared between 100 000 and 100 μg mL^–1^ in methanol. For samples with concentrations below 100 μg mL^–1^ the SIR MS response was used, as the sensitivity of the UV was insufficient. For samples above 100 000 μg mL^–1^, the UV response was used, as this was above the linear range of the SIR MS response. Information on the specific surface area and internal pore structure was obtained from N_2_ adsorption at 77 K on a Micromeritics 3Flex volumetric gas sorption analyzer. Each material (∼10–25 mg) was degassed prior to the experiment (388 K, ∼8 h, 1 × 10^–6^ mbar). Helium was used for free-space determination following isothermal data collection. N_2_ and helium were supplied by Air Liquide and were of purity 99.999%. Pore volume distribution as a function of pore width was calculated from the N_2_ adsorption data using a density functional theory (DFT) fitting and a cylindrical pore – NLDFT Tarazona Esf = 30 K model. The BET surface area was determined following the procedure outlined in ISO 9277.^33^ A Rouquerol correction (for microporous materials) was applied to the BET fitting to calculate surface areas. A resultant correlation function of >0.9999 and a positive intercept were observed for each material (Figure S9).

## 3 Results and Discussion

### 3.1 Characterization of MOFs

**Figure 1:**
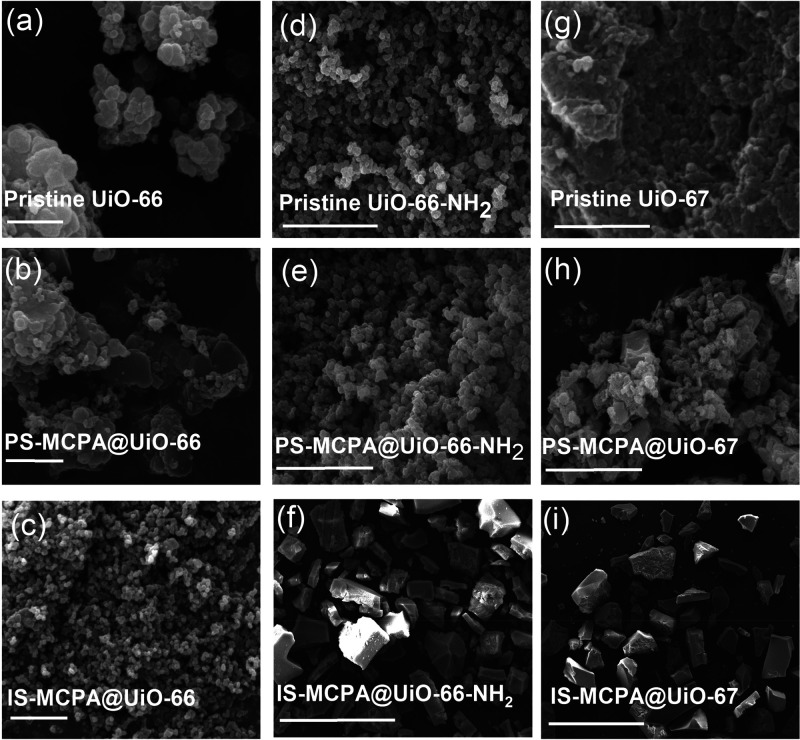
SEM images of three sets of Zr-based MOFs are shown. Scale bars: (a–c) 2 μm, (d, e) 5 μm, (f) 300 μm, (g) 2 μm, (h) 5 μm, and (i) 1 mm.

Three zirconium-based MOFs, UiO-66, UiO-66-NH_2_, and UiO-67 were selected for this study based on their robustness and stability.^31,32^ Two strategies were used to load MCPA into the MOFs: (i) postsynthetic loading, and (ii) in situ loading.^34^ For postsynthetic loading, each MOF was synthesized and activated at 115 °C under a vacuum prior to loading with MCPA. For in situ loading, MCPA was used as a modulator during the synthesis of the MOFs. In the following discussion, the postsynthetically loaded MOFs are referred as PS-MCPA@UiO-66, PS-MCPA@UiO-66-NH_2_, and PS-MCPA@UiO-67, and the in situ loaded MOFs are referred as IS-MCPA@UiO-66, IS-MCPA@UiO-66-NH_2_, and IS-MCPA@UiO-67, respectively. All the pristine and loaded MOFs (Figure 1) were characterized using PXRD, IR, and TGA. The release of MCPA for each of the loaded MOFs was studied over 72 h in both water and ethanol, with amounts of MCPA released quantified via HPLC.

Particle morphologies were examined using scanning electron microscopy. As shown in Figure 1, in situ loaded MOFs produced more homogeneous and larger crystals when compared to their pristine counterparts. Although postsynthetic loading did not show any apparent effect on the particle morphologies for PS-MCPA@UiO-66 and PS-MCPA@UiO-66-NH_2_, a significant change in appearance was observed for PS-MCPA@UiO-67.

**Figure 2:**
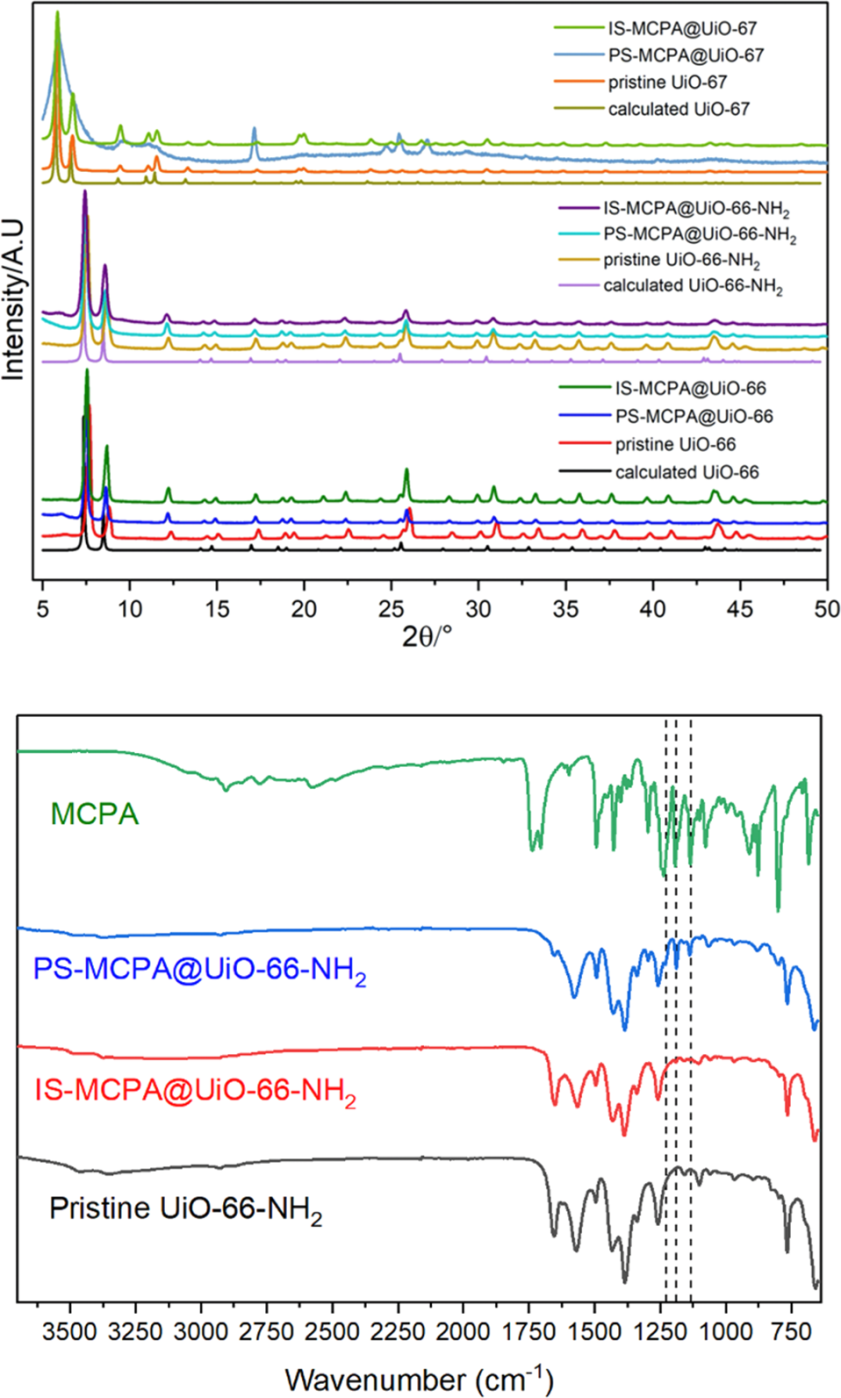
PXRD for all three sets of Zr-MOFs are shown (top). IR spectra for MCPA, PS-MCPA@UiO-66-NH_2_, IS-MCPA@UiO-66-NH_2_, and Pristine UiO-66-NH_2_ are shown (bottom) with the characteristic peak of MCPA at 1230 cm^–1^ present in all loaded samples, indicating the presence of MCPA in these three samples.

The phase purity of the pristine UiO-66, UiO-66-NH_2_, and UiO-67 was confirmed by comparing their PXRD patterns with calculated patterns from the reported single-crystal X-ray diffraction data.^35^ XRD patterns for MCPA-modulated IS-MCPA@UiO-66, IS-MCPA@UiO-66-NH_2_ and IS-MCPA@UiO-67 as well as the postsynthetically loaded MOFs were also compared to the PXRD patterns of their respective pristine MOFs to verify phase purity. With the exception of PS-MCPA@UiO-67, all in situ and postsynthetically loaded MOFs exhibited characteristic Bragg reflection peaks consistent with those reported in literature.^35^ PS-MCPA@UiO-67 exhibited poorer crystallinity, indicative of structural changes that might have occurred during the postsynthetic loading process, as shown in Figure 2. Similar structural degradation of UiO-67 MOFs has been reported in other studies during postsynthetic drug loading.^36,37^

FT-IR spectra (Figure 2 and Figures S1 and S2) for pristine MOFs are consistent with literature.^38,39^ The bands centered around 1670 and 1400 cm^–1^ for synthesized UiO-66 and UiO-67 refer to the symmetric C=O and carboxylate group stretching. Bands centered around 1501 and 1590 cm^–1^ can be attributed to the C=C stretching vibrations of the phenyl ring. For UiO-66-NH_2_ characteristic bands due to the presence of the amine group of the 2-aminoterephthalic acid linker can be seen at 1257 and 1384 cm^–1^ due to the bond stretching between the aryl carbon and the nitrogen of the amine group. Broad peaks at 3464 and 3350 cm^–1^ can be attributed to the symmetric and asymmetric N–H bond stretching, respectively.

**Figure 3:**
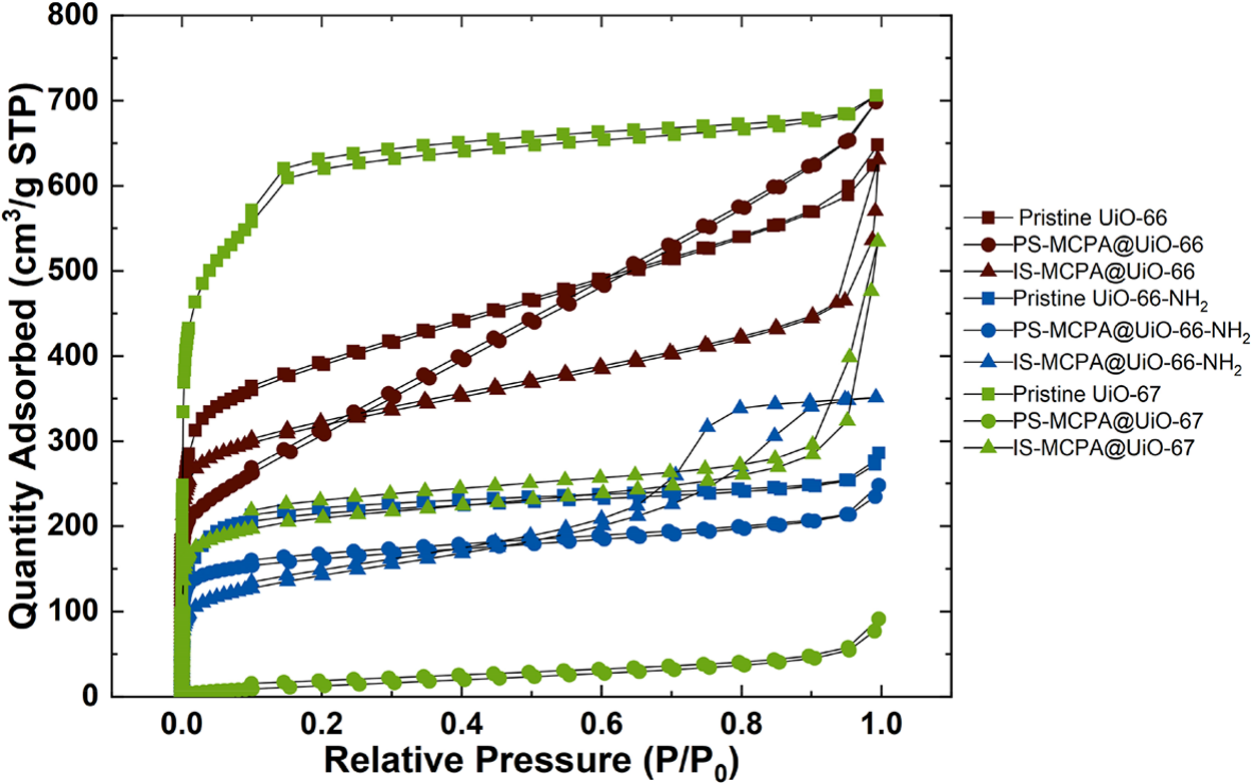
Nitrogen adsorption–desorption isothermal cycles of MOF samples collected at 77 K.

**Figure 4:**
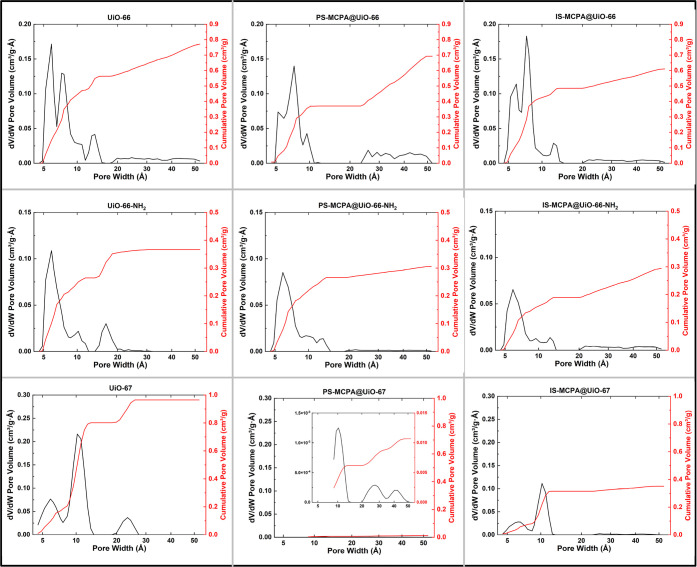
Pore size distribution and cumulative pore volume of the MOF samples (pristine, postsynthetically loaded, and in situ MCPA-loaded), fitted with a DFT cylindrical Tarazona model.

N_2_ gas sorption experiments were carried out on each material to determine surface area, pore volume and pore size distribution. Adsorption–desorption N_2_ isotherms of the synthesized MOFs are shown in Figure 3. Pore size distributions and cumulative pore volume (Figure 4) were calculated by fitting the isotherms to DFT models, with the Tarazona cylindrical pore NLDFT model achieving the best goodness of fit.

As classified by IUPAC,^40^ samples PS-MCPA@UiO66-NH_2_ and UiO-66-NH_2_ each exhibit a classic Type I isotherm, indicating highly microporous materials with limiting uptake governed by micropore volume. This is also reflected in the pore size distribution with limited differential pore volume above 20 Å.

The pristine UiO-67 and IS-MCPA@UiO-67 both exhibit a Type I isotherm with a step at 0.1 and 0.0005 *P*/*P*_o_, respectively. This indicates the presence of different micropores in the sample, as can be seen in the calculated pore size distributions shown in Figure 4, with UiO-67 exhibiting pore sizes 5.9 and 10.2 Å and IS-MCPA@UiO-66 with pore sizes 6.3 and 10.2 Å. IS-MCPA@UiO-67 also exhibits a degree of macropore uptake at higher pressures not revealed in the pore size distribution, which may be due to particulate size and packing from the larger crystallites (see Figure 1(i), creating large interparticulate voids. UiO-66, PS-MCPA@UiO-66 and IS-MCPA@UiO-66 each exhibit a hybrid Type I/II isotherm profile. For these samples a very high nitrogen uptake below 0.03 *P*/*P*_o_ is observed, indicating a high degree of microporosity in the samples followed by a steadily increasing absorption profile indicating unrestricted multilayer formation, likely coming from mesoporosity. This is reflected in the pore size distributions (Figure 4) with an increased level of the cumulative pore volume expressed by mesopores (i.e., ∼50% of PS-MCPA@UiO-66 total pore volume comes from mesopores). IS-MCPA@UiO-66-NH_2_ displays a Type IVa isotherm with a high degree of microporosity and an H2(a) hysteresis at higher pressures, indicating a hierarchical pore structure with capillary condensation in meso or macropores typically >4 nm. Finally, PS-MCPA@UiO-67 exhibits a Type II isotherm with a small degree of microporosity present–suggesting a comparatively nonporous material. This is reflected in the calculated pore size distribution with the very low pore volume found in the sample. The largest surface area was observed for UiO-67 at 2216 m^2^ g^–1^, followed by the pristine UiO-66, with the presence of the NH_2_ functional group for each MOF significantly reducing the available surface area. This can be explained by examining the pore size distributions across the batches. Although UiO-66, PS-MCPA@UiO-66, and IS-MCPA@UiO-66 all exhibit micropores at both ∼5.9 and 7.4 Å (Figure 4), the NH_2_-functionalized samples all showed a significant reduction of pores upon addition of NH_2_ functional group, thus eliminating available pore space and surface area. The functional groups also reduced the level of mesoporosity in the samples, which also affects the total surface area.

PS-MCPA@UiO-67 exhibited the lowest level of adsorption and thus the lowest calculated surface area and pore volume ,indicating the sample was largely nonporous. DFT fitting for pore size distribution did expose some micropores present in the sample but at a very low level per gram (Figure 4). From this observation, and further supported by the XRD data and release studies, it was concluded that the reduced porosity resulted from a significant loss of crystallinity and high loading of the herbicide.

Thermal stability of all MOFs was studied in detail, and the TGA plots are shown in Figures S3– S5. All MOFs were dried in a vacuum oven for 24 h at 115 °C prior to conducting TGA. For UiO-66 MOFs, two stages of weight loss can be observed: the first stage spanning from 100 °C to about 450 °C can be attributed to the dehydroxylation of zirconium oxo-clusters,^41^ whereas the second weight loss occurring at ca. 500 °C is due to the degradation of the linkers and the decomposition of the framework.^42^ For UiO-66-NH_2_ MOFs, weight loss occurs at a range from 100 °C to around 450 °C, which is due to the breaking down of the Zr-oxo/hydroxo clusters.^43^ A second weight loss can be seen at around 550 °C, indicating the decomposition of the MOF due to linker degradation. UiO-67 MOFs show a two step weight loss with the final thermal degradation being at around 525 °C.^44^

### 3.2 Herbicide Adsorption

FT-IR, pore volume analysis, and mass spectrometry all indicated the successful loading of MCPA into the synthesized MOFs. IR spectra of PS-MCPA@UiO-66, PS-MCPA@UiO-67, and PS-MCPA@UiO-66-NH_2_ (Figure 2, Figures S1 and S2) all showed peaks consistent with the presence of MCPA. For example, the presence of the peak at 1230 cm^–1^ originating from C–O stretching bands of MCPA indicates its presence in the loaded MOFs (Figure 2). Some noticeable differences in the IR spectra of PS-MCPA@UiO-66-NH_2_ are observed. A reduction in relative intensity of the peak at 1657 cm^–1^ was observed, which is attributed to the involvement of its linker in the enhancement of adsorption of MCPA.^45^

Pore volume and surface area analyses indicate a significant reduction in available pore volume and BET surface area for postsynthetically loaded MOFs as well as in situ synthesized MOFs compared to their pristine counterparts. DFT calculated pore volumes of UiO-66 (Table 1) decreased upon the postsynthetic loading of MCPA, from 0.772 to 0.693 cm^3^ g^–1^ indicating adsorption of the herbicide. For UiO-66-NH_2,_ the post synthetically loaded sample showed a pore volume reduction of 16%, from 0.366 to 0.306 cm^3^ g^–1^_._ In general, MCPA-modulated MOFs exhibited lower pore volumes and BET surface areas. This can be attributed to either the number of defects introduced into the structure when MCPA was used as a modulator and/or the loading of the herbicide into the pores.^46−49^ PS-MCPA@UiO-67 exhibited the lowest amount of N_2_ adsorption, indicating that the sample has little to no porosity, which is further indicated by the structural changes and loss of crystallinity observed in the PXRD.

**Table 1:** Calculated Pore Characteristics of the MOF Samples

sample	BET surface area (m^2^/g)	pore volume^a^ (cm^3^/g)	% reduction of pore volume^b^
UiO-66	1456 ± 2	0.77	
PS-MCPA@UiO-66	1100 ± 2	0.70	11
IS-MCPA@UiO-66	1195 ± 1	0.61	10
UiO-66-NH_2_	865 ± 6	0.37	
PS-MCPA@UiO-66-NH_2_	619 ± 1	0.31	16
IS-MCPA@UiO-66-NH_2_	509 ± 1	0.29	20
UiO-67	2,216 ± 3	0.96	
PS-MCPA@UiO-67	58 ± 1	0.01	99
IS-MCPA@UiO-67	782 ± 1	0.35	64

a Single point adsorption volume taken at *P*/*P*_0_ 0.94.

b With respect to the pristine MOFs.

Mass spectrometry was used to confirm the adsorption of the herbicide by the MOFs. The fragmentation peak at *m*/*z* 199 confirms the release of MCPA as shown in Figure S6. The release of MCPA was studied in more detail using HPLC.

### 3.3 Polycaprolactone-MOF Composites

**Figure 5:**
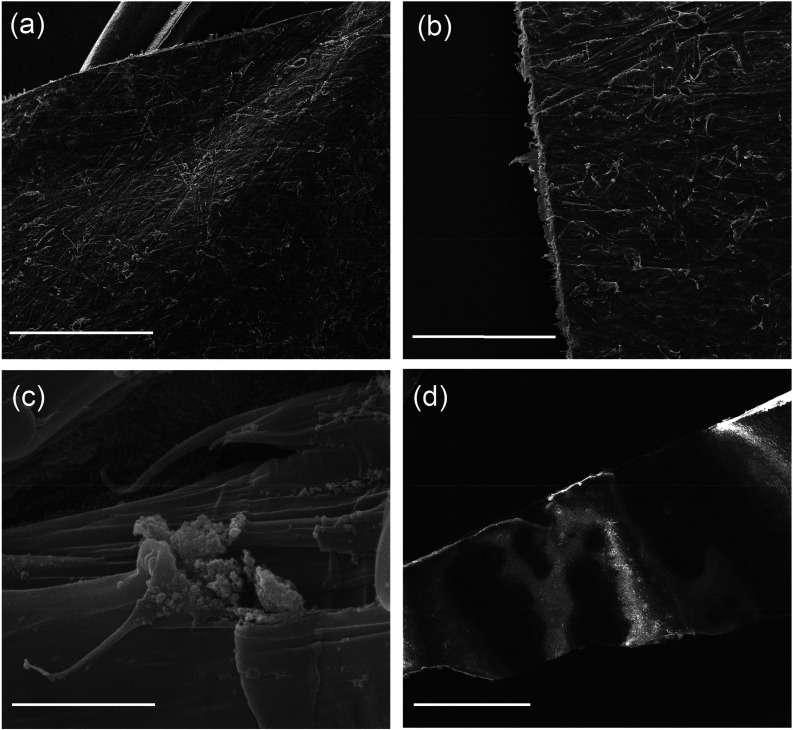
SEM images of gold-sputtered (a) PCL@IS-MCPA@UiO-66, (b) edge of PCL@IS-MCPA@UiO-66, (c) IS-MCPA@UiO-66 MOF particles incorporated into PCL@IS-MCPA@UiO-66, and (d) uncoated SEM image of PCL@PS-MCPA@UiO-66-NH_2_ composite are shown. Scale bars: (a, d) 1 mm, (b) 500 μm, and (c) 5 μm.

**Figure 6:**
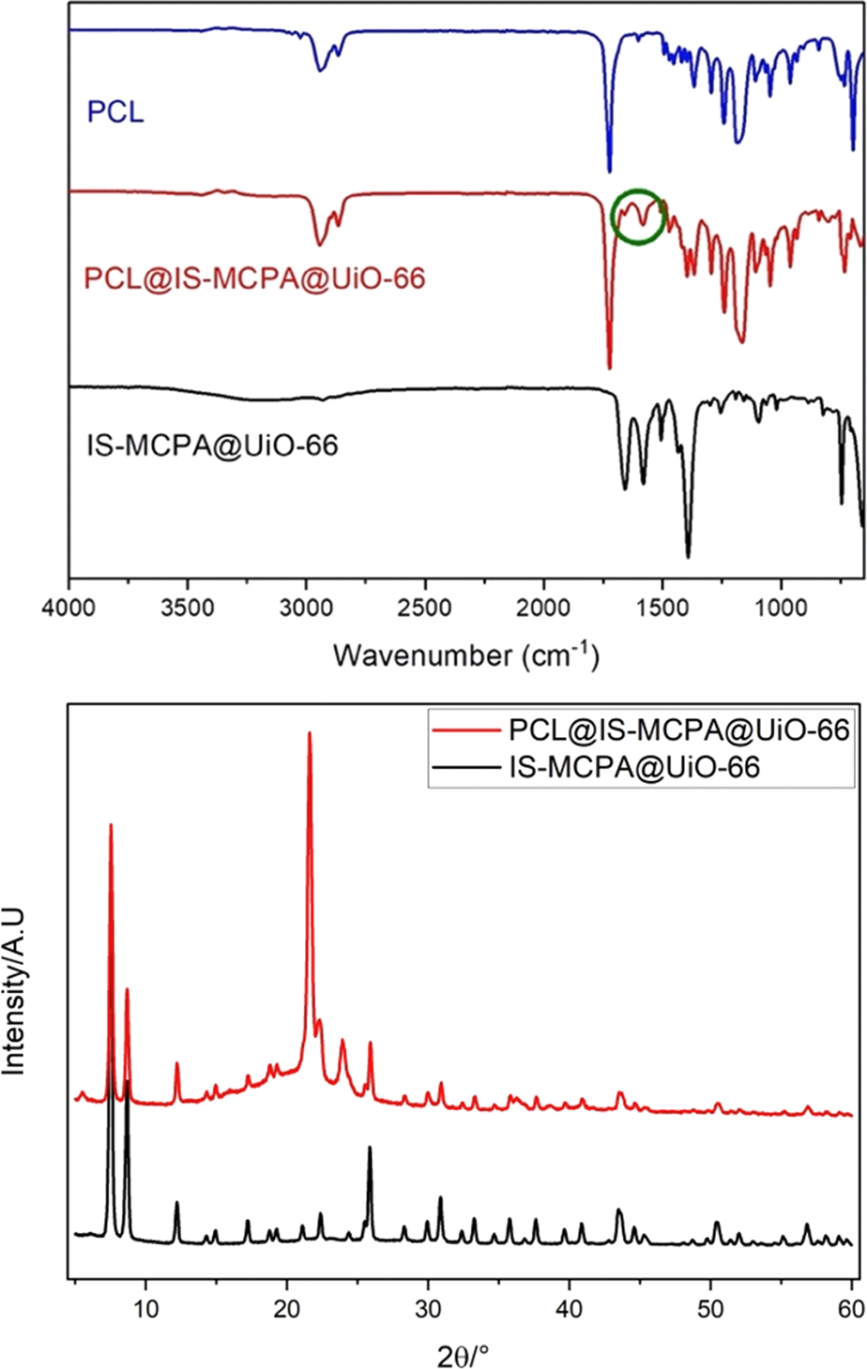
Infrared spectra for PCL, PCL@IS-MCPA@UiO-66, and IS-MCPA@UiO-66 are shown with bands originating from the carboxylate group of the MOFs within the PCL polymer are shown in the green circle (top). Comparison of PXRD plots for PCL@IS-MCPA@UiO-66 and IS-MCPA@UiO-66 is shown (bottom).

The PCL-MOF composites (Figure 5) were characterized using PXRD, TGA, FT-IR, SEM and EDX analyses. PXRD patterns (Figure 6) of the composites show characteristic peaks of the MOFs, confirming that no phase changes occurred during the preparation of the composites. Diffraction peaks at 22 and 25° (2Θ) correspond to the crystallinity of the polymer structure for the PCL composite of IS-MCPA@UiO-66 (referred to as PCL@IS-MCPA@UiO-66).^50^

SEM imaging of the composites showed a scattered distribution of particulate microcrystalline MOFs in the polymer matrix (Figure 5). This is further supported by the elemental mapping using EDX (Figure S7) which shows the Zr is well-dispersed across the composites. In addition, elemental mapping of Cl indicated the incorporation of MCPA within the MOF structure. FT-IR analyses performed on the composites show characteristic peaks at 2941 cm^–1^ for C–C bond stretching and at 1791 cm^–1^ originating from the C=O stretching of PCL. Bands originating from the carboxylate group of the MOFs within the PCL polymer are shown in Figure 6.

TGA for PCL@PS-MCPA@UiO-66 showed a significant loss in weight percentage(87.75%) starting at approximately 220 °C because of the degradation of PCL.^51^ A second small weight loss could be observed at around 514 °C, accounting for the degradation of PS-MCPA@UiO-66, until it reached 4.3% residual weight of inorganic zirconium oxide at 600 °C. From the thermal analysis, we can conclude that the MOF-to-polymer percentage is approximately 6.99%, which is consistent with the theoretical percentage of 6.97% based on synthesis of the composite.

### 3.4 Herbicide Release

All sets of MCPA-loaded MOFs and their PCL composites were left in distilled water and ethanol at room temperature for 72 h. The MOFs and composites were then filtered out and the solvents were analyzed using HPLC to quantify the amount of MCPA released (Figure S9). From the data, it was evident that the postsynthetically loaded MOFs are released with a greater amount of MCPA in both solvents, with PS-MCPA@UiO-66-NH_2_ showing the highest concentration of MCPA in distilled water with 0.037 mg mL^–1^. This can be attributed to the presence of the amine group, which forms a weak hydrogen bond with the carboxylate group present in MCPA. The release of MCPA from PS-MCPA@UiO-67 was the highest in ethanol, at 0.075 mg mL^–1^. However, this higher release is possibly due to its amorphous structure, where the herbicide molecules are loosely held and not contained deep within the lattice.^52^ Encapsulation of MOFs in PCL enhanced the release in distilled water compared to the MOFs alone. For example, the concentration of herbicide released from 20 mg of PCL@PS-MCPA@UiO-66 (containing 1.4 mg of MOF in the composite) was 0.043 mg mL^–1^ compared to 0.019 mg mL^–1^ from 5 mg of PS-MCPA@UiO-66. Similar observations can be noted for PCL@PS-MCPA@UiO-66-NH_2_, where the release for the composite in water was 0.056 mg mL^–1^ compared to a release of 0.037 for PS-MCPA@UiO-66-NH_2_. Observation in earlier studies suggest the burst release from biodegradable polymer matrices is often responsible for such high concentrations, and surface erosion might enhance the effect of burst release.^53^ One other contributing factor to this burst effect might be the release of MCPA into the polymer matrix during the preparation of the composites, resulting in a heterogeneous distribution of herbicide in the composite.

**Figure 7:**
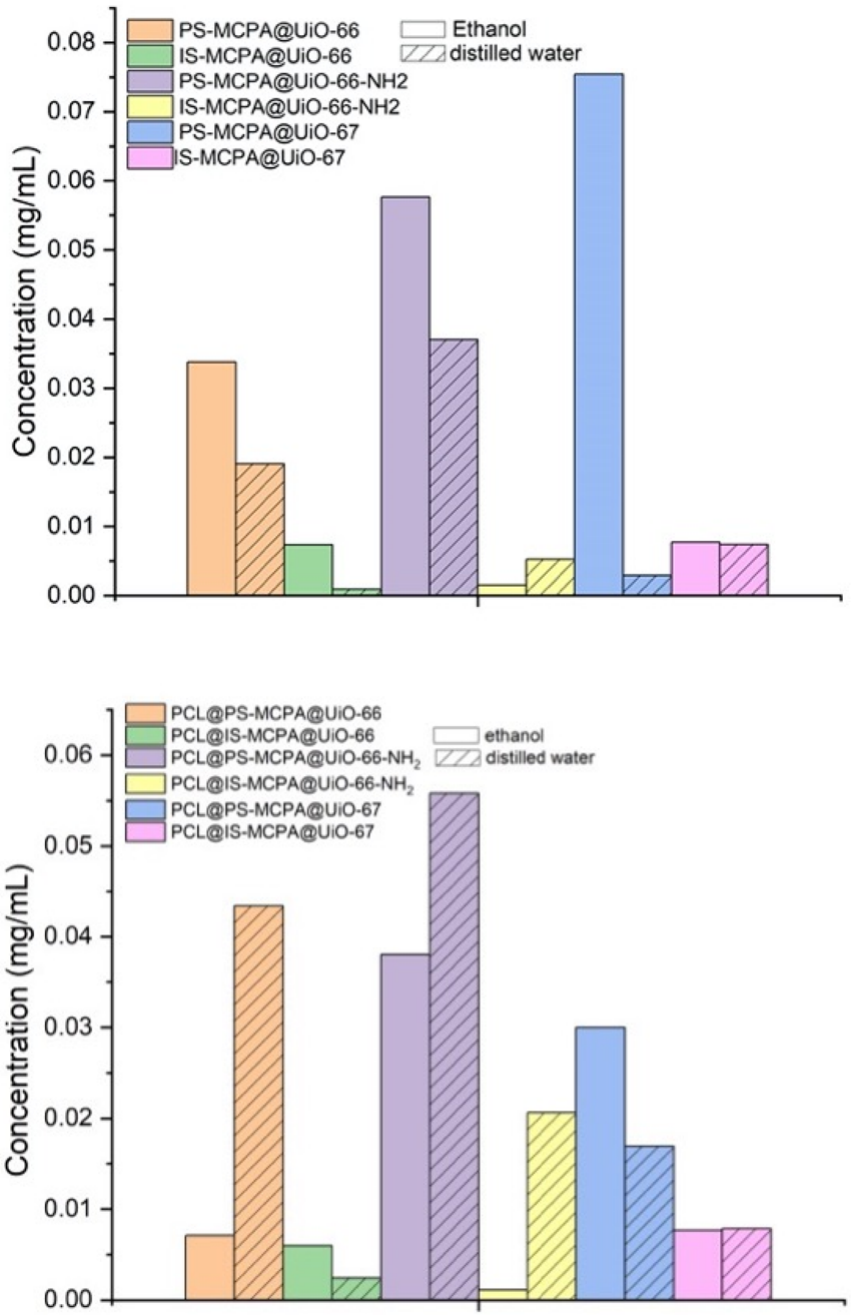
MCPA concentrations released in 72 h for MOFs (top) and MOF-PCL composites (bottom) in ethanol and distilled water.

When comparing the release of MCPA from the polymer composites in both solvents as shown in Figure 7, it was observed that the release was much greater in distilled water than in ethanol. For example, the release of MCPA for PCL@PS-MCPA@UiO-66-NH_2_ into ethanol was 0.038 mg mL^–1^ compared to 0.056 mg mL^–1^ in distilled water. This can be explained by the enhanced release of MCPA due to swelling and possible hydrolytic degradation of the polymer resulting in the surface erosion of PCL in water.^36,54^

Earlier studies showed an LC_50_ of 1.58 mg L^–1^ for the efficacy of MCPA on the broad-leafed plant *S. alba*.^55^ Other studies showed concentrations varying from 0.17 to 6701 mg L^–1^ affecting various growth measurements, from root inhibition, to germination for different types of plants.^56^ In this work, the studied MOFs and their hybrid PCL composites provide us a with range of MCPA concentrations that would result in the inhibition of growth for a diverse range of plants, and therefore, they can be a potential platform for developing an efficient and environmentally friendly way of contact-based pesticide delivery for future agricultural use.

## Conclusions

In this study, we incorporated the herbicide MCPA into three Zr-based MOFs (UiO-66, UiO-66-NH_2_, and UiO-67) using postsynthetic loading and in situ loading during the synthesis of MOFs. The MOFs were characterized in detail confirming successful synthesis and loading. Additionally, it was also observed that the use of MCPA as a modulator during in situ loading resulted in better crystallinity for all three MOFs studied. These MOFs were integrated into a biodegradable polymer (PCL) and release of MCPA from the MOFs and their PCL composites were studied. PS-MCPA@UiO-66-NH_2_ was found to show the highest release of MCPA among the MOFs, and PCL- PS-MCPA@UiO-66-NH_2_ showed the highest MCPA release among the composites. Although the release from PS-MCPA@UiO-67 was among the highest, the high release was attributed to a burst effect caused by the amorphization of its structure. It was also found that the incorporation of the MOFs into PCL tended to enhance the release of MCPA in water. The observed concentrations of the herbicide released provides us with various range of concentrations that can be utilized for plant growth inhibition of different species at various stages. However, more time-dependent studies are underway to further explore the extended release of herbicides. The current study has successfully used MCPA as a test compound as a proof-of-principle as proposed in our initial objectives, with extension to further agrochemical delivery applications as our logical next step.

## Supporting Information Available

Infrared spectra, TGA plots, ESI-MS, BET surface area analyses plots and PXRD (PDF)

## Abbreviations

BET: Brunauer–Emmett–Teller
HPLC: high-performance liquid chromatography
IR: infrared
MCPA: 2-methyl-4-chlorophenoxyacetic acid
MOF: metal–organic framework
PXRD: powder X-ray diffraction
SEM: scanning electron microscope
TGA: thermogravimetric analysis
