# Supplementary material for: Zirconium-Based MOFs and Their Biodegradable Polymer Composites for Controlled and Sustainable Delivery of Herbicides
Source: ACS Appl Bio Mater. 2022 Jul 29;5(8):3972–81. doi: 10.1021/acsabm.2c00499 (PMC9382672; doi:10.1021/acsabm.2c00499)
Supplement: Supplementary file 1 — mt2c00499_si_001.pdf [file mt2c00499_si_001.pdf]

Supporting Information

Zirconium-based MOFs and their biodegradable polymer composites for controlled and sustainable delivery of herbicides

Lila A. M. Mahmoud,[a,b] Richard Telford,[a] Tayah C. Livesey,[a] Maria Katsikogianni,[a] Adrian L. Kelly,[c] Lui R. Terry,[d] Valeska P. Ting,[d] and Sanjit Nayak,[a]*

[a]School of Chemistry and Biosciences, University of Bradford, Bradford, BD7 1DP, United Kingdom; Corresponding author: s.nayak@bradford.ac.uk

[b]School of Pharmacy, Al-Zaytoonah University of Jordan, Amman 11733, Jordan

[c]Polymer IRC, Faculty of Engineering and Informatics, University of Bradford, Bradford, BD7 1DP, United Kingdom

[d]Bristol Composites Institute, Department of Mechanical Engineering, University of Bristol, BS8 1TR United Kingdom

Contents:

Figure S1. FT-IR for UiO-66 set

Figure S2. FT-IR for UiO-67 set

Figure S3.TGA plots of UiO-66 set

Figure S4.TGA plots of UiO-66-NH_2 set

Figure S5.TGA plots of UiO-67 set

Figure S6. Mass spectroscopy for PS-MCPA@UiO-66 in Ethanol over 24 hours

Figure S7. EDX images of composite PCL@IS-MCPA@UiO-66

Figure S8.TGA for PCL@PS-MCPA@UiO-66

Figure S9. BET Plot of each MOF sample, displaying correlation function, slope, Y-intercept and C values.

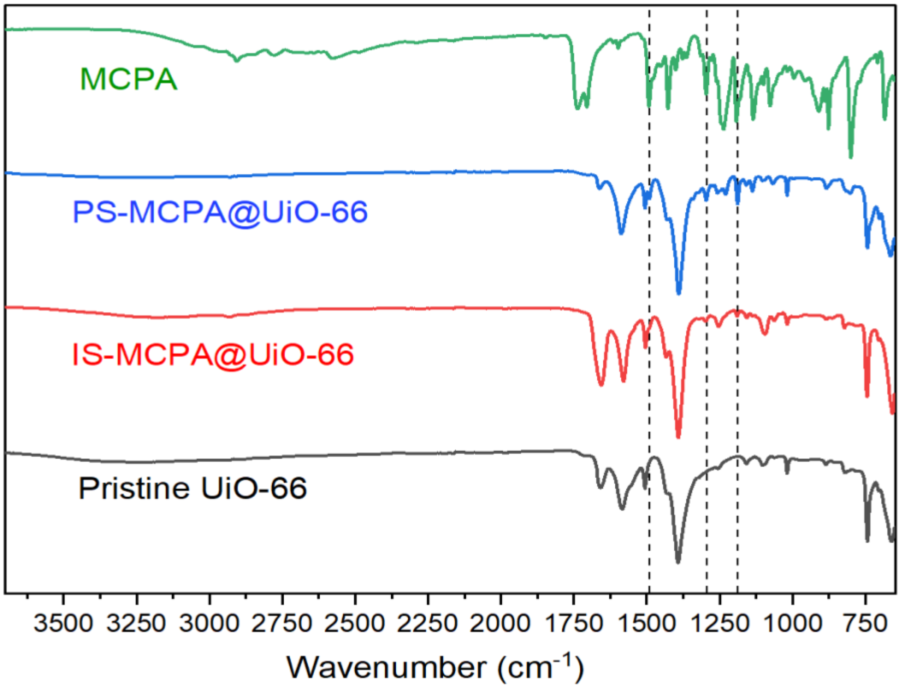

Figure S1. FT-IR for UiO-66, PS-MCPA@UiO-66 and IS-MCPA@UiO-66

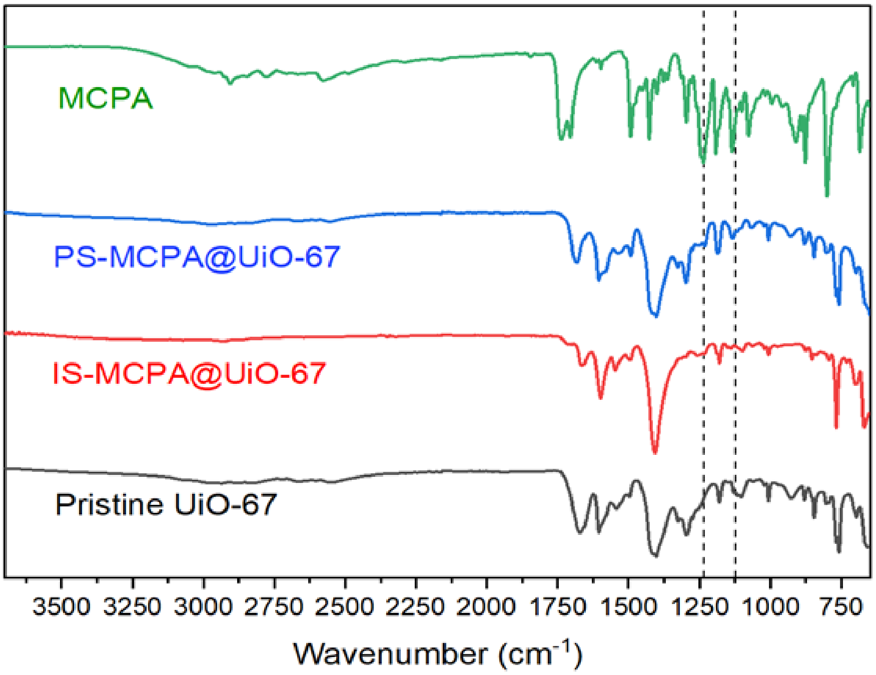

Figure S2. FT-IR for UiO-67, PS-MCPA@UiO-67 and IS-MCPA@UiO-67

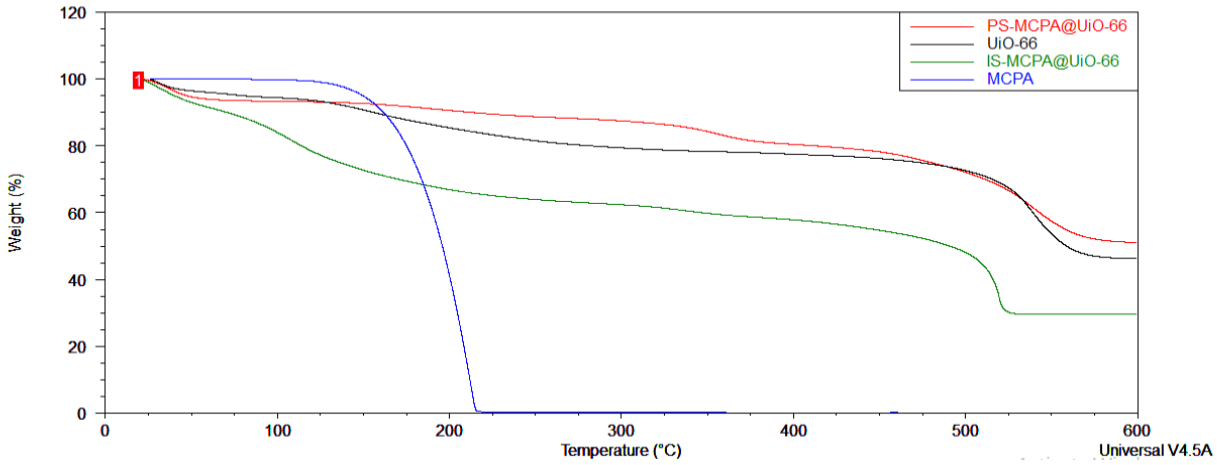

Figure S3. TGA plot for UiO-66, PS-MCPA@UiO-66 and IS-MCPA@UiO-66

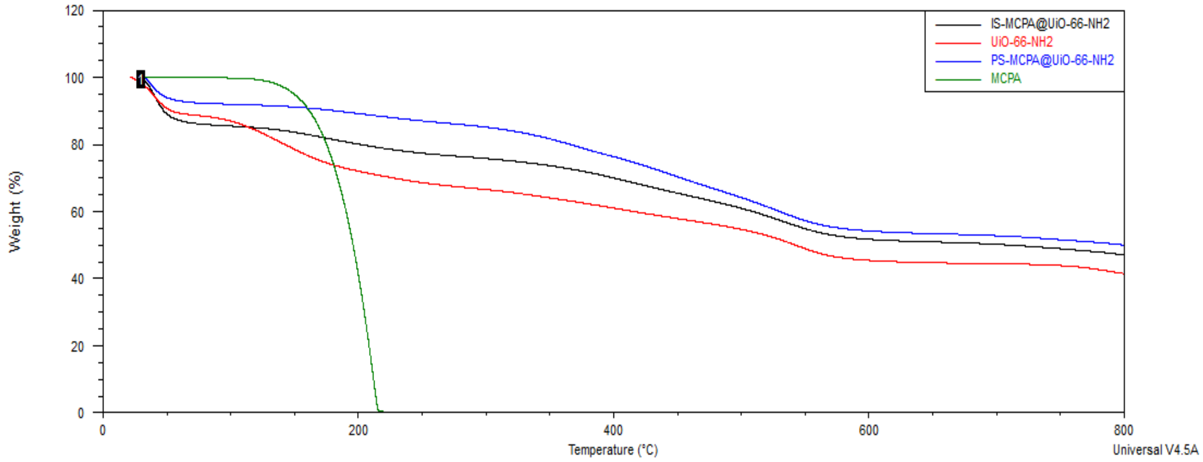

Figure S4. TGA plot for UiO-66-NH_2, PS-MCPA@UiO-66-NH_2 and IS-MCPA@UiO-66-NH_2

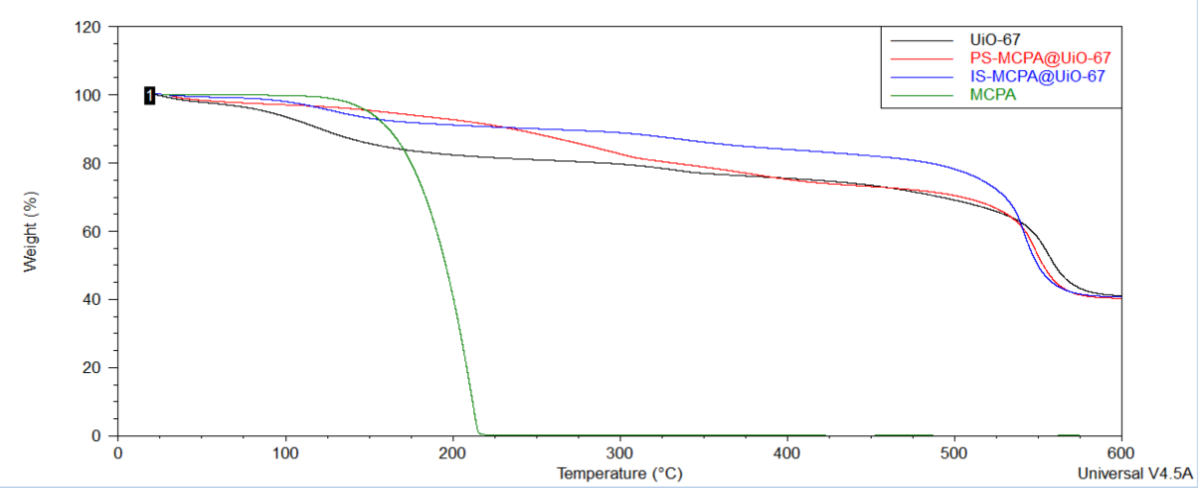

Figure S5. TGA plot for UiO-67, PS-MCPA@UiO-67 and IS-MCPA@UiO-67

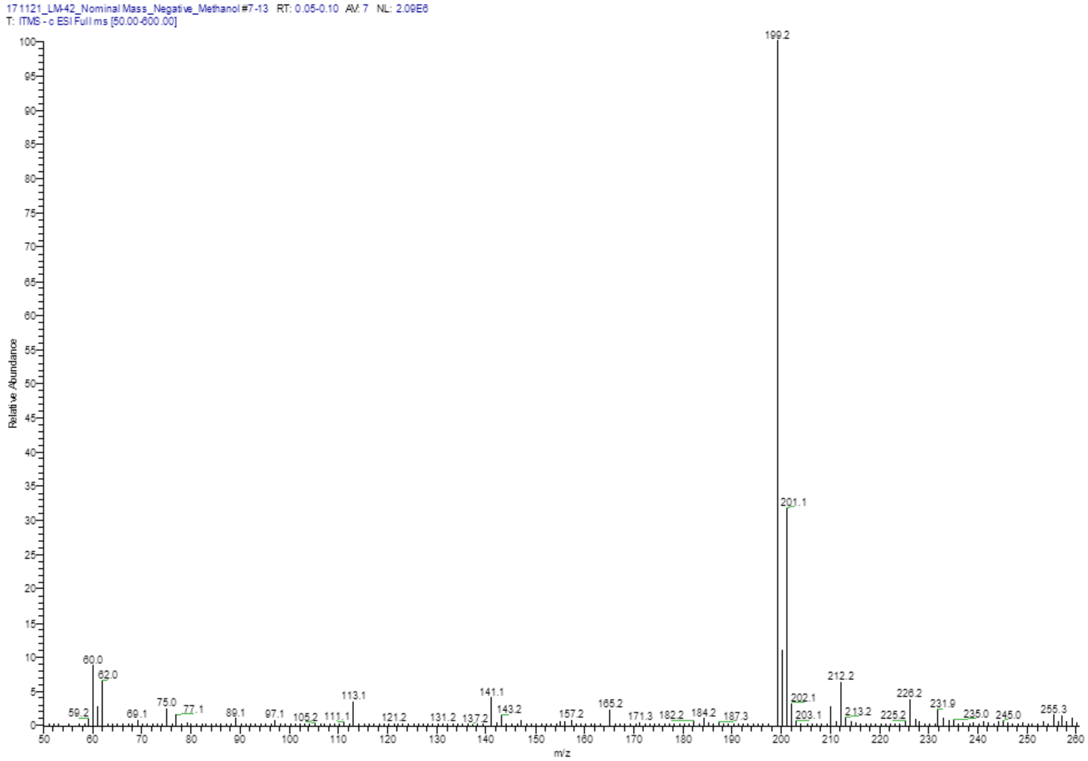

Figure S6. Mass spectroscopy for PS-MCPA@UiO-66 in ethanol over 24 hours

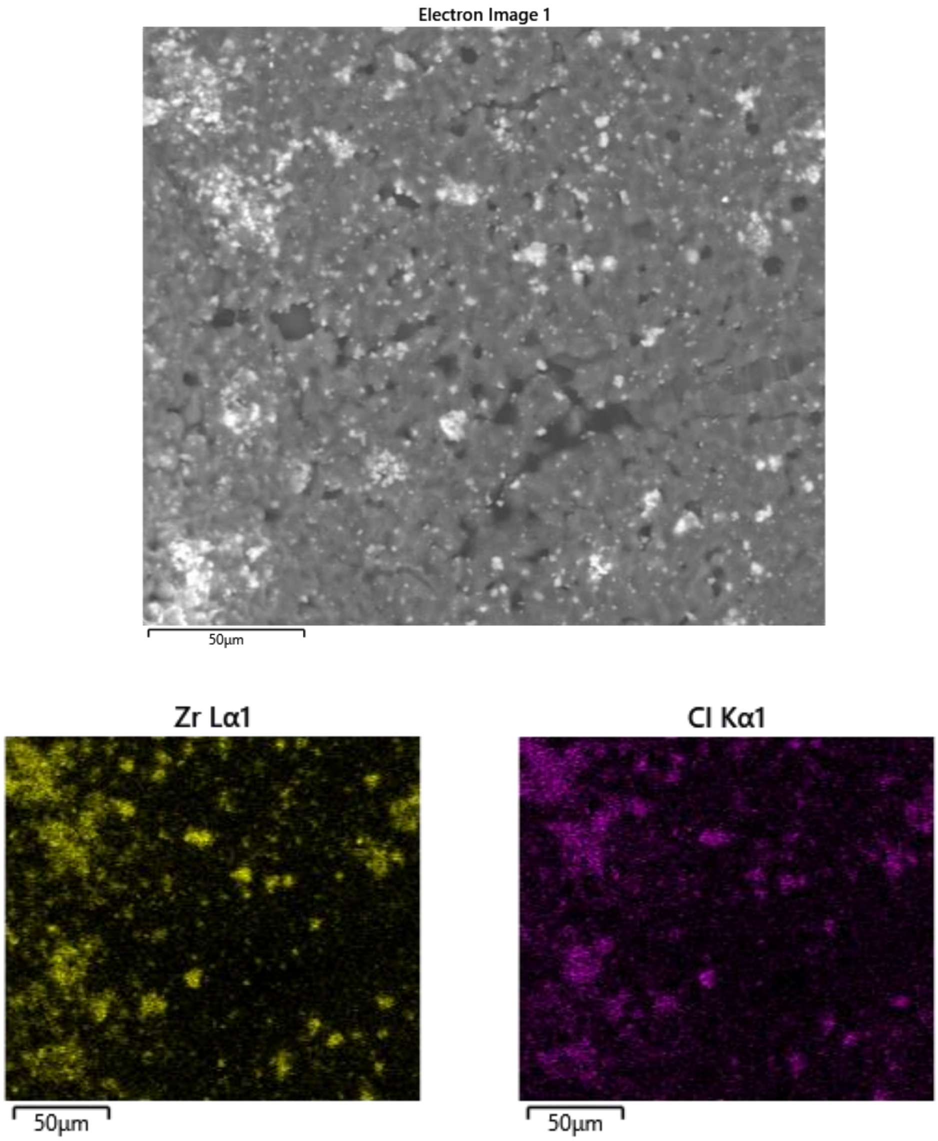

Figure S7. EDX images of composite PCL@IS-MCPA@UiO-66

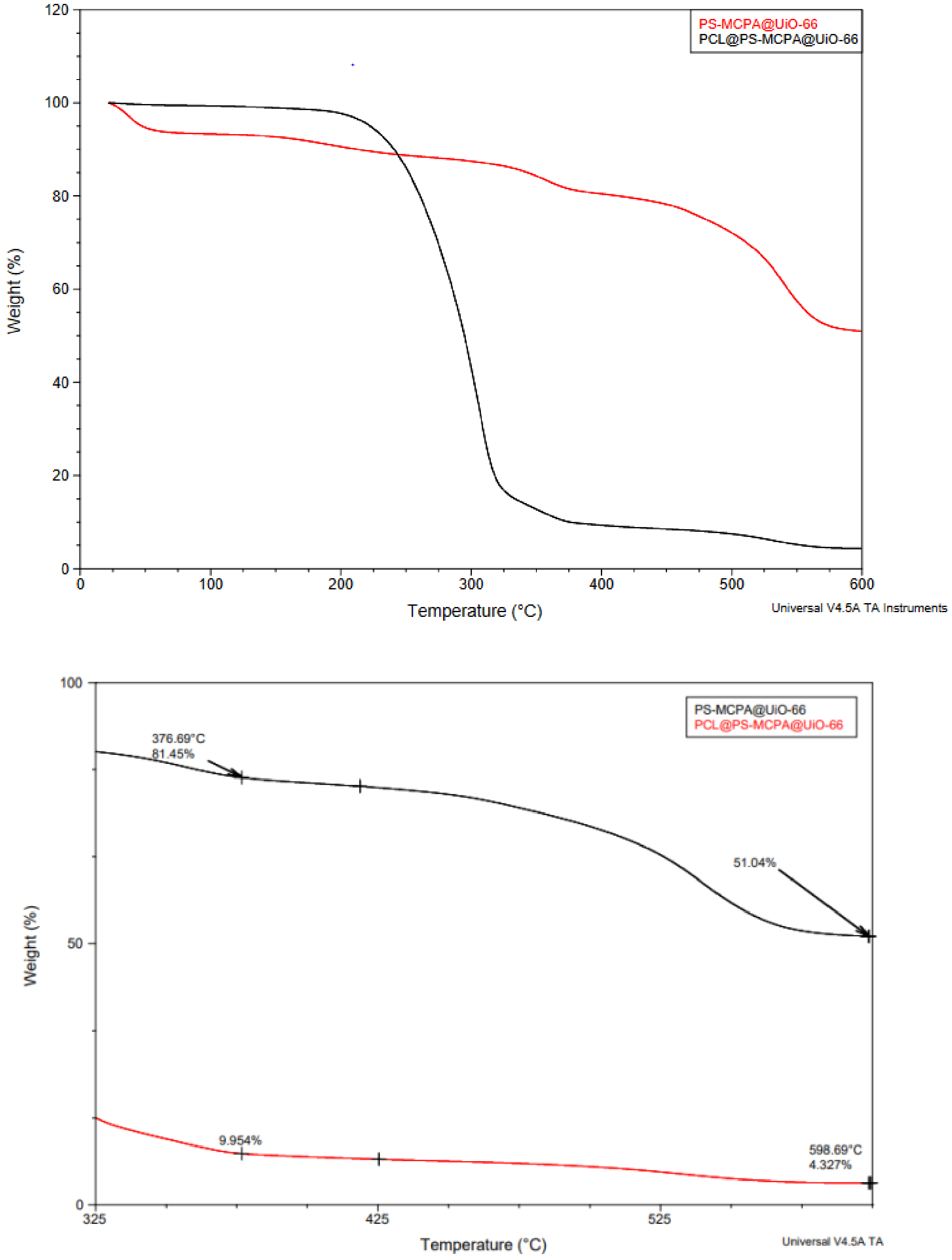

Figure S8. TGA for PCL@PS-MCPA@UiO-66

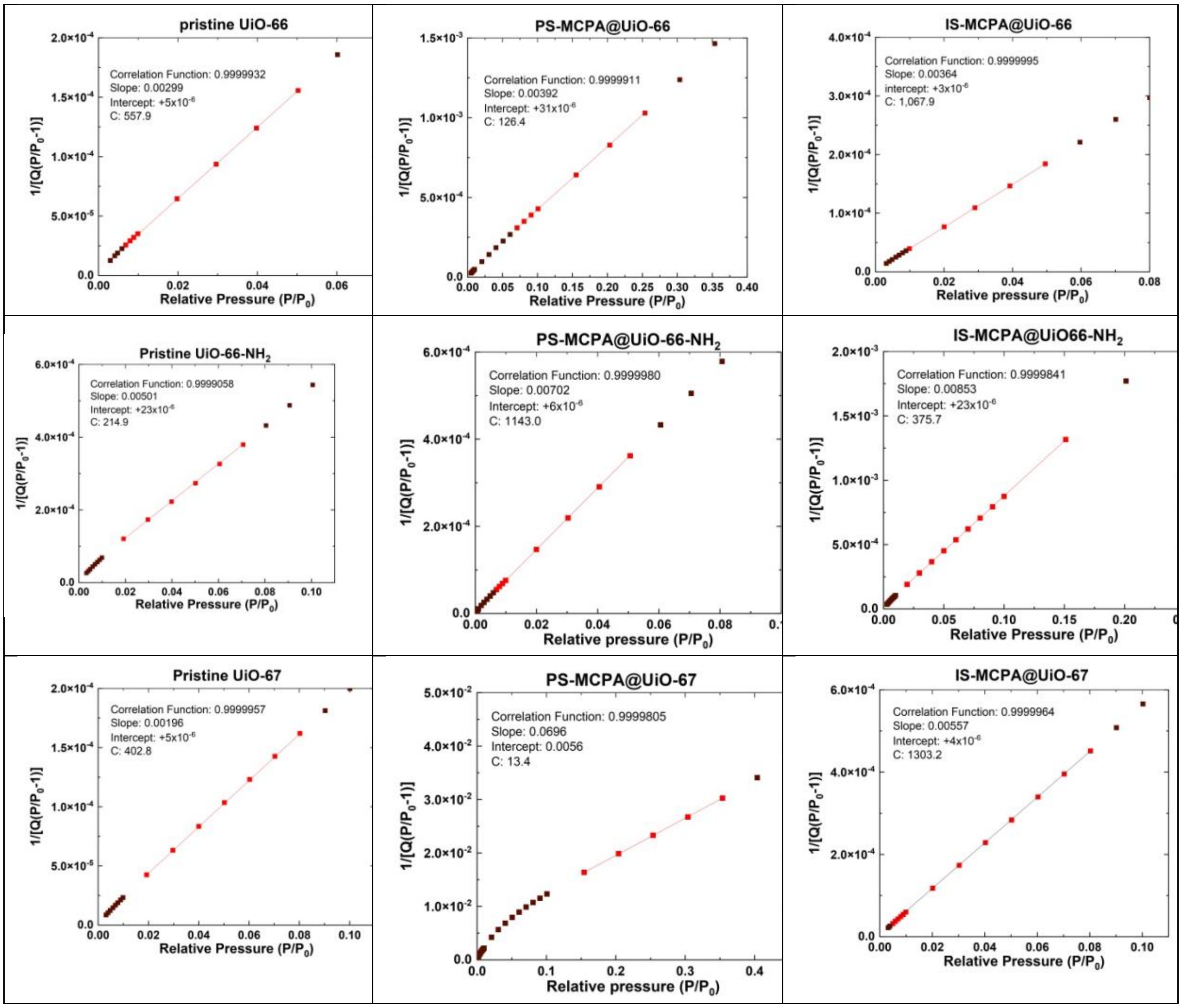

Figure S9. BET Plot of each MOF sample, displaying correlation function, slope, Y-intercept and C values.
